# A CASE OF MULTIPLE MYELOMA PRESENTING AS A BULLOUS DERMATOSIS

**DOI:** 10.4103/0019-5154.41653

**Published:** 2008

**Authors:** Ülker Gül, Arzu Kiliç, Müzeyyen Gönül, Seray Külcü Çakmak, Aylin Okçu Heper

**Affiliations:** *From Ankara Numune Education and Research Hospital, 2^nd^ Dermatology Clinic, Ankara, Turkey*; 1 *From Ankara University School of Medicine, Department of Pathology, Ankara, Turkey*

**Keywords:** *Bullous dermatosis*, *multiple myeloma*, *skin*

## Abstract

Multiple myeloma is a malignant plasma cell proliferative disorder that produces a monoclonal immunoglobulin protein. The skin involvement and the development of bullous disease are rarely seen features in multiple myeloma. We present a 55-year-old man with a longstanding, large, tense bullous eruption and hypertrophic scars over his body accompanied recently with weight loss and fatique. He had no response to the previous treatments, which included oral glucocorticoids and dapsone. Histologic examination of the lesions revealed subepidermal bullae, while no immunoflourescence staining was observed. In a further detailed labarotory examination, multiple myeloma was detected. After the treatment of multiple myeloma with chemotherapy, the lesions regressed. Patients with longstanding, recurrent, unusual bullous eruption should be investigated for the development of multiple myeloma.

## Introduction

Multiple myeloma (MM) is a malignancy of neoplastic plasma cells that generally produces a monoclonal immunoglobulin protein.^1^,^2^ The skin involvement and the development of bullous disease are rarely seen in MM.^2^ In this report, we present a case of MM presenting as an unusual bullous dermatosis.

A 55-year-old man presented with a vesiculobullous eruption of 5-year duration and consequent development of hypertrophic scars. He described an increase in eruption for the last two months accompanied with a marked weight loss. He denied using any treatments since two years.

**Fig. 1:**
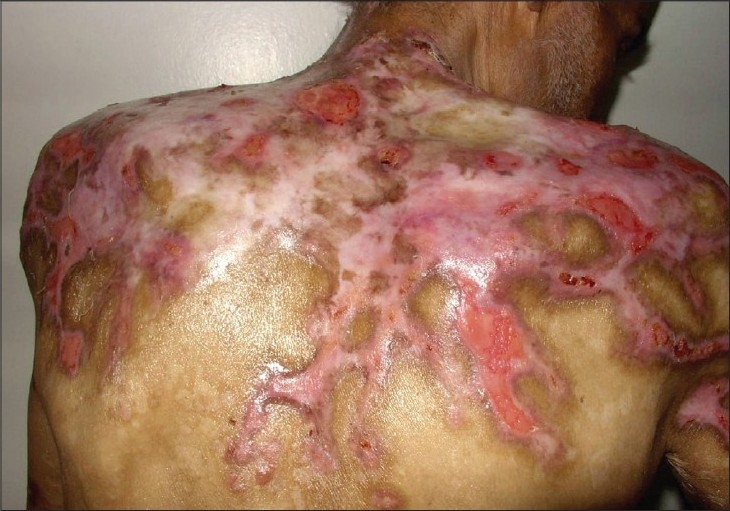
Eroded areas localized on shoulders are seen

Physical examination was normal except for enlarged lymph nodes on both axillary and inguinal regions. Dermatological examination revealed vesicles and bullaes on the trunk, sacrum and lower extremities (Fig. 1). No vesicles or eroded areas were observed in the oral mucosa. Nikolsky's sign was negative.

Histopathological examination of the bullous lesions revealed subepidermal bullae. Direct immunofluorescence findings were negative.

**Fig. 2:**
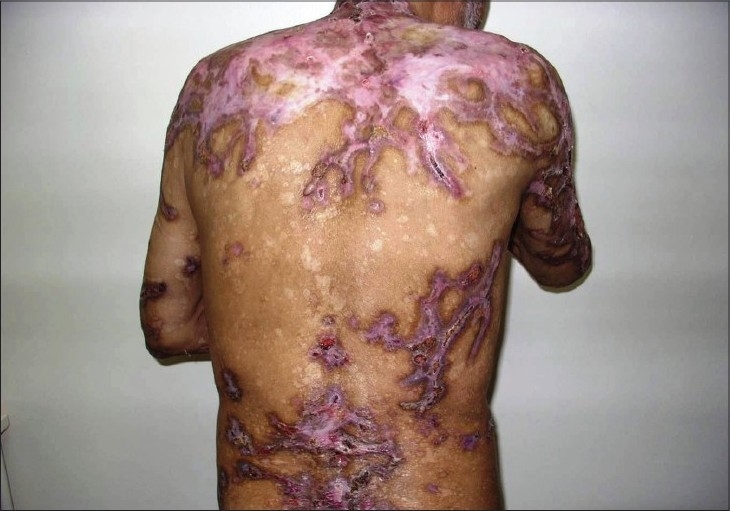
After the treatment of multiple myeloma, eroded lesions completely regressed and only hypertrophic scars are seen

The abnormal laboratory examination results were as follows: hemoglobin 6.9 g/dl (N: 14-17.5 g/dl), hematocrite 22% (N: 42-50%), platelet 515.000/mm^3^ (N: 150-450/mm^3^), albumin 30 g/L (N: 35-54 g/L). The erythrocyte sedimentation rate was elevated. X-ray graphics of the lung and bones and the abdominopelvic ultrasonographic examination were also normal. Protein electrophoresis showed a significant spike in the gamma region with a value of 46.3 g/L (normal 7-16 g/L). Serum immunofixation electrophoresis was consistent with IgG monoclonal gammopathy, and urine immunofixation test showed lambda light chain. Aspiration biopsy and biopsy of bone marrow revealed 15-20% plasma cells. The patient was consulted with Hematology Department, and he was diagnosed as having MM. Chemotherapy consisting of vincristine, dexametasone and adriamycine was started. After the first cycle, the patient's eruption regressed completely (Fig. 2).

MM has a wide spectrum ranging from monoclonal gammopathy of unknown significance (MGUS) to plasma cell leukemia.^1^,^3^ It has been reported that at least some of the MM cases develop from MGUS.^3^

Skin involvement in MM is relatively rare.^2^,^4^ There have been reported cases of MM associated with dermatological disorders like pyoderma gangrenosum, Sweet's syndrome, erythema elevatum diutinum, panniculitis, necrobiotic xanthogranuloma, acquired cutis laxa, follicular hyperkeratosis, POEMS syndrome, telengiectasia macularis eruptiva perstans, toxic epidermal necrolysis, erythema multiforme, subcorneal pustular dermatosis, and Grover's disease.^1^–^4^

There are also reports that indicate the association of unusual bullous disorders and MM.^4^,^5^ In most of these cases, bullous dermatoses do not meet the histological and immunological criteria of known bullous disorders. Myeloma protein can possess the antibody activity against basal membrane antigens. Thus, the destruction of basement membrane zone and bullae formation occurs.^4^

Our patient had a longstanding bullous disease that was resistant to various therapies. Bullous lesions regressed completely by the end of the first cycle of the chemotherapy. We think of the possibility that he might have had MGUS, and we would like to emphasize that patients with longstanding and unusual bullous diseases should be examined thoroughly for underlying MM and/or MGUS.
